# Glutamate involvement in calcium–dependent migration of astrocytoma cells

**DOI:** 10.1186/1475-2867-14-42

**Published:** 2014-05-19

**Authors:** Abdelkader Hamadi, Grégory Giannone, Kenneth Takeda, Philippe Rondé

**Affiliations:** 1 Laboratoire de Biophotonique et Pharmacologie, CNRS, UMR 7213, Université de Strasbourg, Illkirch 67401, France; 2 Interdisciplinary Institute for Neuroscience and UMR CNRS 5297, University of Bordeaux, Bordeaux 33000, France

**Keywords:** Glutamate release, Migration, U-87MG cell, Calcium spikes

## Abstract

**Background:**

Astrocytoma are known to have altered glutamate machinery that results in the release of large amounts of glutamate into the extracellular space but the precise role of glutamate in favoring cancer processes has not yet been fully established. Several studies suggested that glutamate might provoke active killing of neurons thereby producing space for cancer cells to proliferate and migrate. Previously, we observed that calcium promotes disassembly of integrin-containing focal adhesions in astrocytoma, thus providing a link between calcium signaling and cell migration. The aim of this study was to determine how calcium signaling and glutamate transmission cooperate to promote enhanced astrocytoma migration.

**Methods:**

The wound-healing model was used to assay migration of human U87MG astrocytoma cells and allowed to monitor calcium signaling during the migration process. The effect of glutamate on calcium signaling was evaluated together with the amount of glutamate released by astrocytoma during cell migration.

**Results:**

We observed that glutamate stimulates motility in serum-starved cells, whereas in the presence of serum, inhibitors of glutamate receptors reduce migration. Migration speed was also reduced in presence of an intracellular calcium chelator. During migration, cells displayed spontaneous Ca^2+^ transients. L-THA, an inhibitor of glutamate re-uptake increased the frequency of Ca^2+^ oscillations in oscillating cells and induced Ca^2+^ oscillations in quiescent cells. The frequency of migration-associated Ca^2+^ oscillations was reduced by prior incubation with glutamate receptor antagonists or with an anti-β1 integrin antibody. Application of glutamate induced increases in internal free Ca^2+^ concentration ([Ca^2+^]_i_). Finally we found that compounds known to increase [Ca^2+^]_i_ in astrocytomas such as thapsigagin, ionomycin or the metabotropic glutamate receptor agonist t-ACPD, are able to induce glutamate release.

**Conclusion:**

Our data demonstrate that glutamate increases migration speed in astrocytoma cells via enhancement of migration-associated Ca^2+^ oscillations that in turn induce glutamate secretion via an autocrine mechanism. Thus, glutamate receptors are further validated as potential targets for astrocytoma cancer therapy.

## Background

Primary brain neoplasm derived from glial cells account for more than 40% of all brain tumors. Among gliomas, astrocytomas represent the most common type of glial tumors and are generally associated with poor prognosis as these tumor cells often diffusely infiltrate neighboring brain structures by migrating along defined pathways such as blood vessels or myelinated nerves. This characteristic makes surgical resection rarely efficient because by the time the primary tumor can be removed, secondary tumors may have already invaded the surrounding parenchyma. Hence, the aggressiveness of astrocytomas could be decreased by inhibiting cell migration, thereby confining the tumor in its original location.

Migration is a cellular process by which motile cells interact with different adhesion molecules presented by other cell types and extracellular matrix. Binding of adhesion proteins to their receptors generates signals that regulate cell proliferation and migration. A change in calcium homeostasis has been shown to represent one of the major intracellular signals implicated in the multiple and highly coordinated molecular events necessary to promote migration. For example, oscillations of intracellular Ca^2+^ modulate neuronal migration of growth cones [1,2] and cerebellar granule cells [3]. Changes in intracellular Ca^2+^ have been reported to be responsible for persistent forward migration of neutrophils [4]. Several signaling pathways can be implicated in Ca^2+^ signaling observed during migration, including those mediated by adhesion receptors of the integrin family and those mediated by serum which could promote activation of the MAP kinase cascade [5,6]. Hence, in mouse fibroblasts, integrin engagement leads to phosphorylation of FAK and the subsequent conformation change promotes direct activation of PLC-γ1 with the FAK autophosphorylation site Tyr-397, resulting in the generation of IP_3_ and release of Ca^2+^ from internal Ca^2+^ stores [7]. Previously, we demonstrated that in astroctytoma, increases in [Ca^2+^]_i_ are associated with increased phosphorylation of FAK at Tyr-397 [8].

The initial promoter of the Ca^2+^ signal appears to be cell type specific. In fish keratinocytes, integrin-dependent cell motion stimulates stretch-activated Ca^2+^-channels [9] whereas in arteriolar smooth muscle, integrin ligands modulate L-type Ca^2+^ channels [10]. In the developing brain, migration of immature neurons to their final destination is correlated with the expression of both N-type Ca^2+^ channels and glutamate receptors. Moreover, the rate of movement of granule cells appears to be controlled by the activity of NMDA receptors [11]. In mice, glutamate serves as a chemoattractant for neurons in the developing cortex, signaling cells to migrate into the cortical plate via NMDA receptor activation [12]. In astrocytes, pharmacological blockade of NMDA receptors inhibits PSA-NCAM biosynthesis and dramatically diminishes cell migration from neurohypophyseal explants [13]. Nevertheless, the precise role of glutamate in mediating cell migration is not well understood, especially for glioma cells. For example, it has been described that glioma release large amounts of glutamate via both compromised glutamate transporters [14] and the cystine-glutamate exchange system Xc^-^[15]. The pathophysiological significance of elevated glutamate in the extracellular space has not been fully investigated, although it has been suggested that it might promote active neuronal cell death, thereby creating space for the growing tumor to expand [16] and enhancing glioma migration via activation of Ca^2+^-permeant AMPA receptors [17].

In this study, we investigated the role of glutamate in favoring glioma cell migration. We demonstrate that the human astrocytoma cell line U87MG is able to release glutamate in the extracellular space which in turn, activates glutamate receptors in an autocrine/paracrine manner, thus leading to calcium signaling involved in both cell migration and enhanced glutamate release.

## Results

### Glutamate-enhanced migration of astrocytoma cells

**Figure 1:**
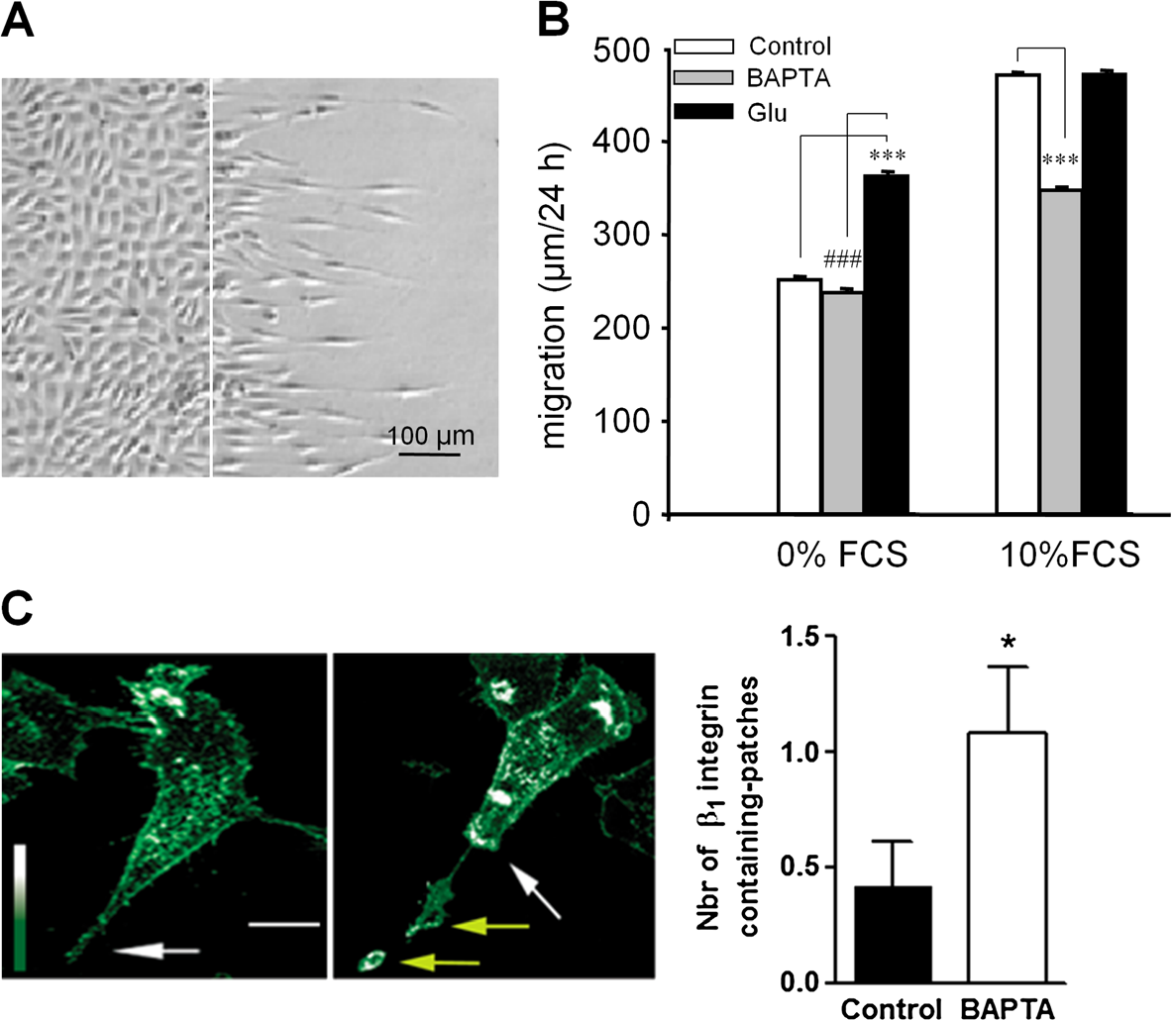
**Glutamate increases motility in astrocytoma cells. A**: Representative field showing U-87MG cells plated on Matrigel-coated dishes after 24 hours of migration in a wound healing model of migration (the white line indicates the border of the lesion). **B**: The speed of migration of U-87MG cells plated on matrigel-coated dishes was measured 24 h after lesion. The presence of 10% fetal calf serum (FCS) in the culture medium increases the rate of migration by 51%. Serum-dependent migration is reduced by 55% when the cells were first loaded for 30 minutes with 20 μM BAPTA-AM to chelate intracellular Ca^2+^. Glutamate increased serum-independent migration while serum-dependent migration was left unchanged. Data are mean ± SEM; n = 4 independent experiments with 120 to 240 cells analyzed per condition; ***p < 0.001. **C**: Cells loaded or not with 20 μM BAPTA, were allowed to migrate on Matrigel-coated glass coverslips and then immunostained with the anti-β_1_ integrin antibody. White arrows indicate the cellular tail and yellow arrows, patches of β_1_ integrin. Scale bar, 20 μm. The number of patches of β_1_ integrin-containing structures found at the rear of the cell was quantified in control and BAPTA-loaded cells. Data are mean ± SEM of 3 independent experiments with 4 fields analyzed per experiment.

Initially, using the wound-healing model of cell migration, we measured the migration speed of U87MG cells plated on matrigel-coated dishes. In the presence of 10% FCS the rate of migration was 470 ± 3 μm/24 h and 251 ± 4 μm/24 h in the absence of serum. Incubating the cells with the cell permeant Ca^2+^ chelator BAPTA/AM reduced serum-dependent migration while serum-independent migration was unchanged. This indicates the existence of a Ca^2+^-dependent migration process mediated at least in part by serum. In the absence of serum, addition of glutamate increased the rate of migration by 44% to 362 ± 3 μm/24 h, whereas in the presence of serum the rate of migration was unchanged by glutamate addition (Figure 1). Taken together, this suggests a role for glutamate and Ca^2+^ signaling in mediating cell motility. The decrease in migration observed for BAPTA-loaded cells likely involves a regulatory mechanism controlling the attachment of integrins to the substratum. We therefore compared the distribution pattern of β_1_ integrins in migrating cells loaded or not with BAPTA. Buffering Ca^2+^ lead to the accumulation of β_1_ integrins at the tail of the cell (Figure 1C). Moreover, patches of integrin-containing structures were found at the rear of the cell, consistent with “ripping release”, as the cell moved forward. This is consistent with changes in Ca^2+^ being necessary to promote the recycling of β_1_ integrins from the tail of the cell.

### Migration of astrocytoma cells is associated with intracellular calcium oscillations

**Figure 2:**
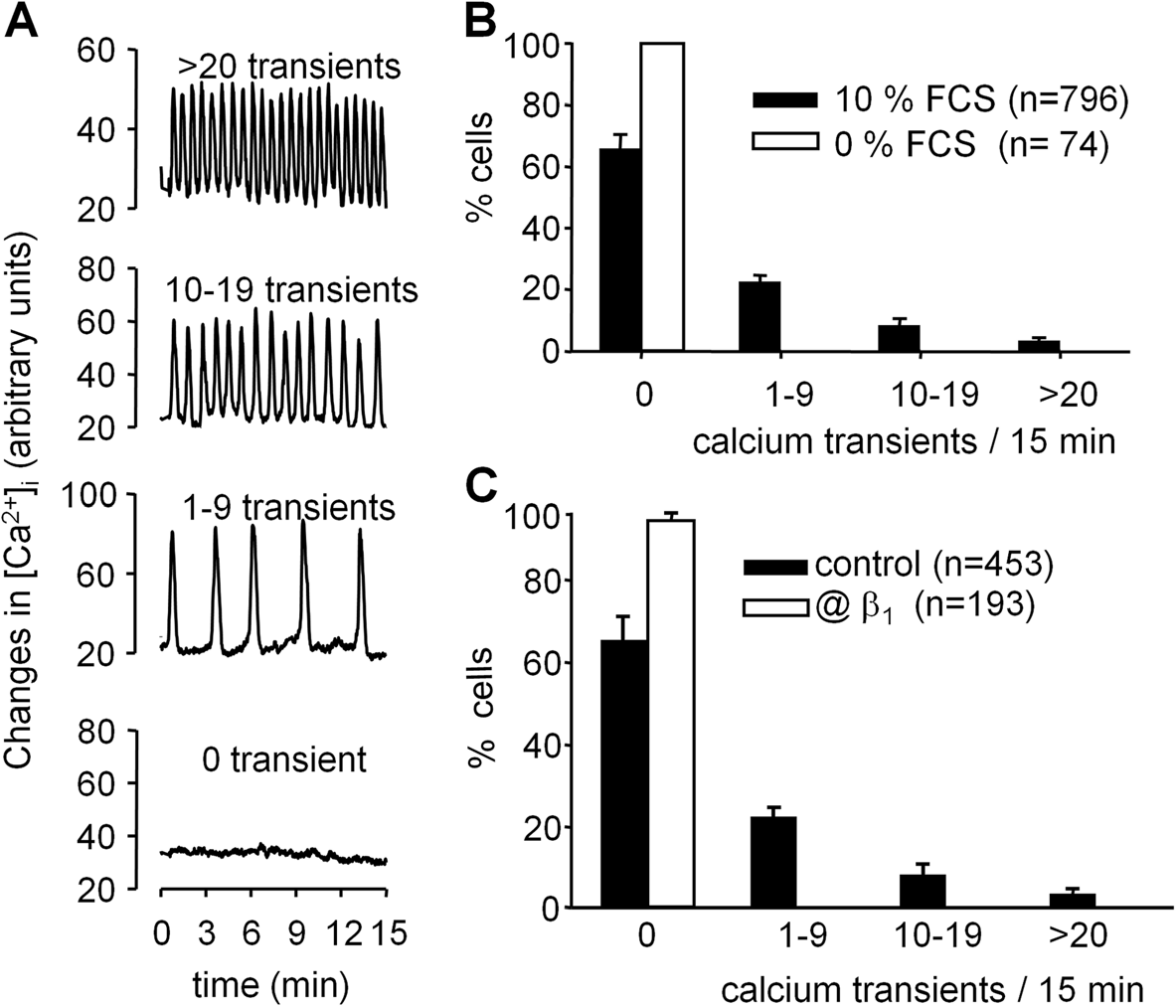
**Migrating astrocytoma cells exhibit serum-dependent intracellular Ca**^**2+ **^**oscillations at varying frequencies. A**: Cells plated on Matrigel-coated glass-coverslips were loaded with Oregon green and observed using a confocal microscope. Representative traces of individual cells show a varying number of spontaneous intracellular Ca^2+^ transients. Cells were grouped according to the number of intracellular Ca^2+^ transients displayed during the 15 minutes acquisition period. **B**: The percentage of cells displaying 0, 1–9, 10–19 and >20 intracellular Ca^2+^ transients during 15 minutes, in the presence or absence of 10% FCS. **C**: The percentage intracellular Ca^2+^ transients during 15 minutes, in the presence or absence of the functional antibody P4C10 directed against β1 integrins. Data are mean ± SEM of 4 independent experiments, n indicate the number of cells analyzed per conditions.

The above results prompted us to further analyze the role of Ca^2+^ in migration. To do so, we used confocal imaging of intracellular Ca^2+^ in single migrating cells. In the presence of serum, 36% of cells displayed intracellular Ca^2+^ oscillations at varying frequencies during the 15 min observation period, whereas no spontaneous variations in Ca^2+^ were detected in the absence of serum (Figure 2A, B). To assess whether calcium oscillations were associated with migration, we tested a function-blocking antibody directed against β_1_ integrins, one of the major integrins present in astrocytoma cells. For this purpose, cells were incubated with the anti-β_1_ antibody P4C10 prior to calcium measurements. In the presence of anti-β_1_ antibody, a large decrease in the percentage of cells displaying Ca^2+^ transients was observed, up to 96%, consistent with an essential role of integrin engagement in the generation of Ca^2+^ oscillations (Figure 2C). Of note, this antibody also significantly decreased the rate of migration of astrocytomas in the presence of serum by 73%, with a mean value of 172 ± 4 μm/24 h (n = 4).

### Ca^2+^-mobilizing agents induce glutamate release from astrocytoma cells

**Figure 3:**
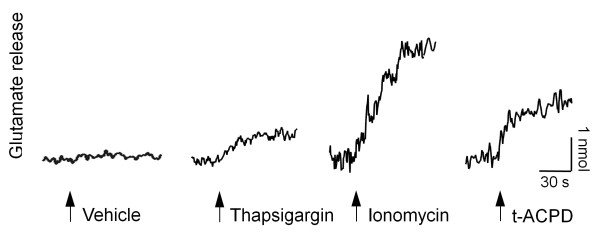
**Ca**^**2+ **^**mobilizing agents induced glutamate release in astrocytoma cells.** Fluorescence traces representing glutamate release elicited by vehicle (far left) thapsigargin (left), an inhibitor of the endoplasmic reticulum Ca^2+^ ATPase, ionomycin (right), a Ca^2+^ ionophore, and t-ACPD (far right), a selective metabotropic glutamate receptor agonist. The traces are representative for 4–6 independent experiments.

It is well described that gliomas and astrocytomas release large amounts of glutamate in the medium as compared to non cancer cells [14]. Moreover, it has been previously shown that glioma invasion may be promoted via an autocrine glutamate signaling loop [17]. The release of glutamate by glioma/astrocytoma cells could be both Ca^2+^-dependent and Ca^2+^-independent. Therefore, as U87MG cell migration is associated with calcium oscillations and augmented in the presence of glutamate, we tested whether compounds known to increase [Ca^2+^]_*i*_ were able to induce release of glutamate from U87MG cells. For this purpose, we used an enzymatic assay to continuously monitor the release of glutamate in migrating cells plated on matrigel-coated coverslips in order to keep the same experimental conditions as those used to measure the speed of migration and changes in [Ca^2+^]_*i*_. We first used two compounds, thapsigagin and ionomycin, known to promote large increases in [Ca^2+^]_*i*_ in these cells [18]. As shown in Figure 3, both thapsigargin and ionomycin were able to produce glutamate release. Moreover, t-ACPD, an agonist of metabotropic glutamate receptors which has been shown to provoke increases in [Ca^2+^]_*i*_ in astrocytes [19,20] also induced glutamate release. On the other hand, we were unable to observed glutamate release using specific agonists of NMDA and AMPA/kainate glutamate receptor subtypes (data not shown).

### Glutamate increases intracellular Ca^2+^ levels

**Figure 4:**
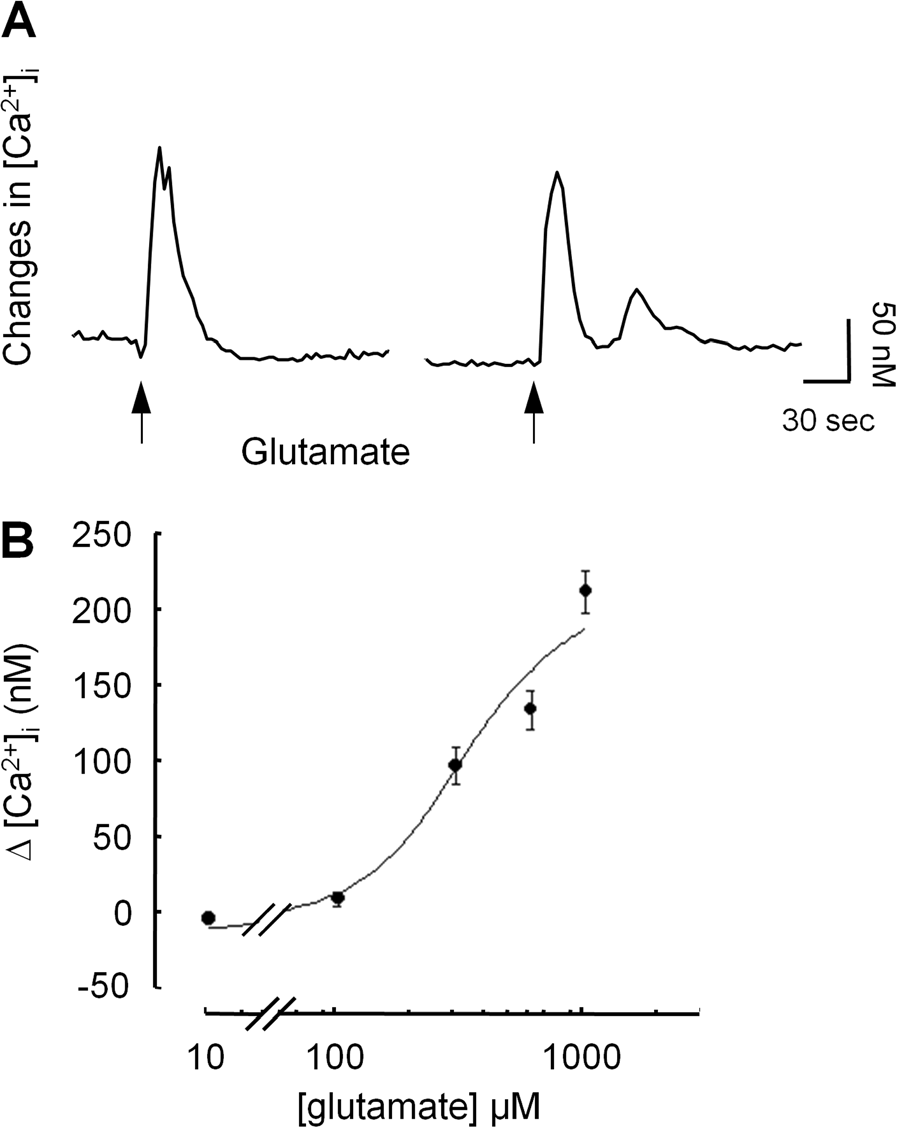
**Glutamate-induced increase in [Ca**^**2+**^**]**_**i **_**in astrocytoma cells. A**: Quantification of changes in [Ca^2+^]_i_ as measured microfluorimetrically in Fura-2 loaded cells. Typical individual responses observed after addition of 300 μM glutamate are shown. In a large number of cells, glutamate elicited single Ca^2+^ spike while a second spike can be observed in 10-20% of the cells analyzed. **B**: Glutamate induced a concentration-dependent increase in [Ca^2+^]_i_. Data are mean ± SEM of 4 independent experiments.

As most glutamate receptors are known to alter calcium homeostasis, we designed experiments to test whether glutamate was involved in migration-associated Ca^2+^ oscillations using Fura-2 imaging of intracellular Ca^2**+**^ in single migrating cells. Addition of glutamate (1 μM-1 mM) in replacement of serum did not mimic the effect of serum as in the majority of the cells, no oscillation of [Ca^2+^]_*i*_ could be detected during the migration process (data not shown). Nevertheless, addition of 300 μM glutamate produced a sharp increase in [Ca^2+^]_*i*_. In 85% of the cells, the increase in [Ca^2+^]_*i*_ resulted in a single transient of Ca^2+^ whereas in the other 15%, oscillations of small amplitude were detected following the initial response (Figure 4A). The increase in [Ca^2+^]_*i*_ was dose-dependent with an EC_50_ of 284 ± 16 μM and a maximum increase of 210 ± 26 nM Ca^2+^ (Figure 4B).

### Glutamate reuptake inhibitor induces increased migration-associated Ca^2+^ oscillations

**Figure 5:**
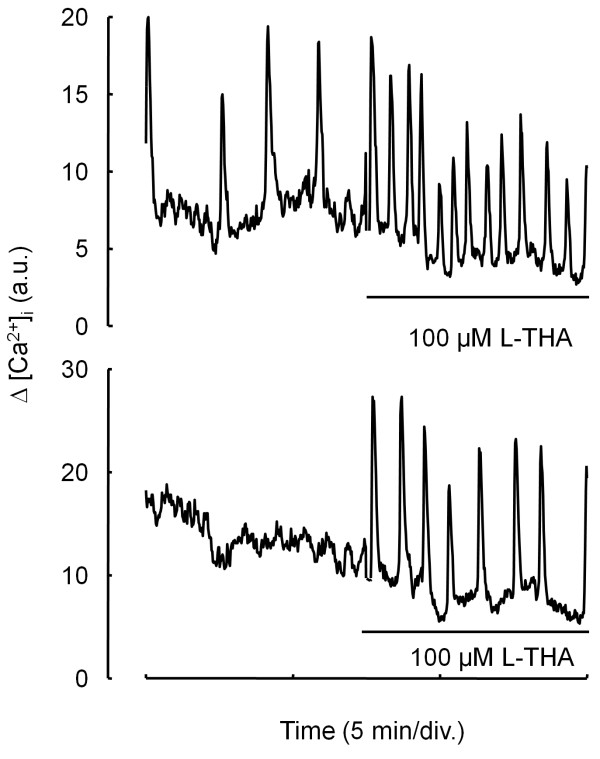
**Inhibition of glutamate reuptake induced increase in Ca**^**2+ **^**oscillations frequency.** Fluo-3 loaded cells were allowed to migrate on Matrigel-coated glass coverslips and changes in fluorescence were measured at 522 nm using confocal microscopy before and after addition of 100 μM L-THA. Top: Typical individual response of spontaneous Ca^2+^ oscillations is presented. Note the increase in spontaneous Ca^2+^ oscillations frequency after addition of L-THA. Bottom: In non oscillating, quiescent cells, addition of L-THA induced Ca^2+^ oscillations as measured by changes in fluo-3 fluorescence. Data are mean ± SEM of 4 independent experiments with 67 cells analyzed per condition.

Because addition of glutamate in the absence of serum did not induce Ca^2+^ oscillations comparable to those observed in the presence of serum, we tested whether glutamate could enhance serum-mediated Ca^2+^ oscillations. As it is difficult to estimate the concentration of glutamate present in the medium, we chose to increase the concentration of glutamate in the extracellular medium by inhibiting the reuptake of glutamate. In agreement with our previous result, in the presence of serum, 36% of the cells displayed intracellular Ca^**2+**^ oscillations at varying frequencies during the 15 min observation period. Addition of 100 μM L (-)-*threo*-3-hydroxyaspartic acid, a potent inhibitor of both glial and neuronal uptake of glutamate [21] produced a two-fold increase in the frequency of Ca^2+^ oscillations. Analyzing in further detail the complex pattern of behavior of L-THA-treated cells, we observed that in 30% of the cells, L-THA had no effect whereas in 39%, L-THA caused an increase in Ca^2+^ oscillation frequency (Figure 5A) and in the remaining 31%, L-THA initiated oscillatory Ca^2+^ behavior in cells which did not display spontaneous variations of [Ca^2+^]_*i*_ before addition of the compound (Figure 5B). Taken together, these results suggest that glutamate present in the serum and/or released by the cells is able to alter Ca^2+^ homeostasis, thereby contributing to enhanced migration.

### Glutamate antagonists reduce migration and migration-associated Ca^2+^ oscillations

**Figure 6:**
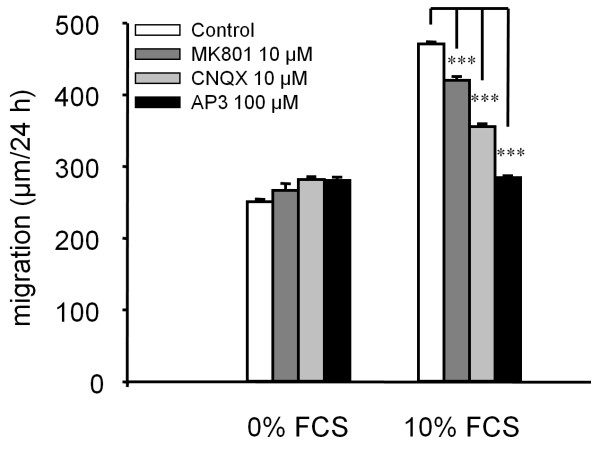
**Glutamate antagonists reduced astrocytoma cell migration.** In the absence of serum, glutamate antagonists had no effect on the speed of migration while in the presence of serum, migration was significantly reduced, the major effect being observed using AP3 an antagonist at metabotropic glutamate receptor. Data are mean ± SEM; n = 4 independent experiments with 50 to 240 cells analyzed per condition; ***p < 0.001.

**Figure 7:**
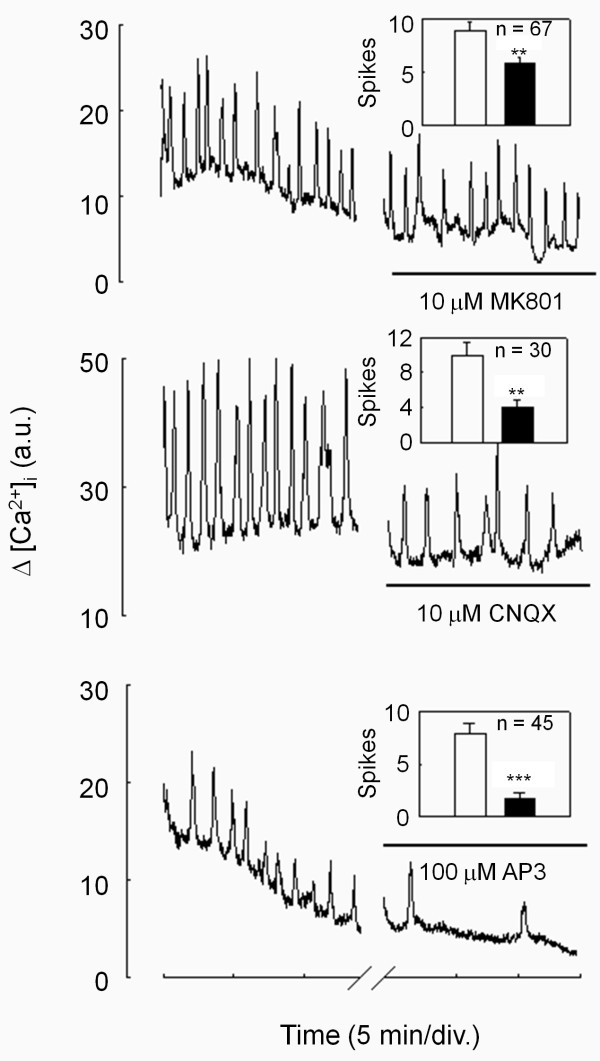
**Glutamate antagonists reduced spontaneous Ca**^**2+ **^**oscillations observed during the migration process.** Typical individual responses of spontaneous Ca^2+^ oscillations before and after addition of glutamate antagonists are presented. In the presence of the ionotropic NMDA glutamate receptor antagonist MK801 (top), or ionotropic AMPA/kainate glutamate receptor antagonist CNQX (middle), or metabotropic glutamate receptor antagonist AP3 (bottom), the frequencies of Ca^2+^ oscillations were reduced. **Insets:** Data are mean ± SEM of the number of Ca^2+^ spikes displayed during a 15 min observation period from 3 to 7 independent experiments with 30 to 67 cells analyzed per condition, **p < 0.005, ***p < 0.0001.

As glutamate increases cell migration and Ca^2+^ oscillation frequency, we tested whether the serum-dependent component of the migration process is mediated at least in part by glutamate acting at glutamate receptors. Selective antagonists at NMDA receptors, MK801 [22], kainate receptor, CNQX [23] and a large spectrum antagonist at metabotropic receptor, AP3 [24] were added in the culture medium supplemented or not with 10% serum after the lesion was achieved. As shown in Figure 6, all antagonists reduced significantly serum-dependent migration. Migration was reduced by 24% in the presence of 10 μM MK801, 53% in the presence of CNQX and 85% in the presence of AP3. On the other hand, all three compounds were without effect on the serum-independent component of migration. This is consistent with glutamate receptors being involved in serum-mediated migration. Next, we determined which type of glutamate receptor was involved in the oscillations of [Ca^2+^]_*i*_ observed during migration. For this purpose, U87MG cells displaying oscillatory behavior were incubated for 30 min with antagonists of various glutamate receptor subtypes and the numbers of Ca^2+^ spikes were compared before and after treatment. Addition of 10 μM MK801 slightly but significantly reduced the number of Ca^2+^ spikes. In contrast, addition of 10 μM CNQX resulted in a 60% inhibition of the number of Ca^2+^ spikes and 100 μM AP3 caused a 78% decrease in Ca^2**+**^ oscillation frequency (Figure 7). The order of potency of these compounds is in agreement with their respective abilities to inhibit serum-mediated migration and highlights the close relationship existing between migration and Ca^2+^ oscillation behavior in these cells [8,25].

## Discussion

In this study, we have demonstrated that glutamate released by human astrocytoma cells contributes to enhanced migration by a mechanism involving glutamate-associated Ca^2+^ oscillations. Indeed, antagonists of glutamate receptors inhibit both cell migration and migration-associated Ca^2+^ oscillations while glutamate itself stimulates migration under serum deprivation. Moreover, the glutamate reuptake inhibitor L-THA increases the frequency of Ca^2+^ oscillations and induces Ca^2+^ oscillations in quiescent cells. These effects can be correlated with the inhibitory action of the Ca^2+^ chelator BAPTA on the migration of these cells.

Ca^2+^-dependent migration was first demonstrated in neutrophils where the speed of migration and persistent forward motion were correlated with intracellular Ca^2+^ levels [26]. In cerebellar microexplant cultures, while a global increase in intracellular Ca^2+^ was not correlated with cell mobility, it was rather found that the frequency and amplitude of Ca^2+^ fluctuations control the rate of migration of granule cells [3]. Moreover, granule cells start their radial migration only after the expression of N-type Ca^2+^ channels and glutamate receptors on the plasmalemmal surface [27] supporting the idea that glutamate receptors associated with Ca^2+^ signaling may be a key component of cellular migration. Similarly, we reported that the migration of smooth muscle cells and U87MG cells were dependent upon oscillations of intracellular Ca^2+^[18,28]. The role of glutamate and Ca^2+^ in regulating proliferation and migration of neurons during development is now well recognized but little is known concerning whether glutamate alters proliferation and migration of tumor cells. Several studies have shown that glutamate antagonists limit tumor growth of various human tumor cells, including astrocytoma [29,30]. The mechanisms implicated in this anti-cancer effect involve both a decrease in tumor cell proliferation and a reduction of cell motility. Interestingly, the anti-proliferative effect of glutamate antagonists was Ca^2+^ dependent and resulted from decreased cell division and increased cell death. Nevertheless, the molecular events involved in the reduction of tumor cell locomotion and invasiveness have not been described. Our study demonstrates that glutamate antagonists limit migration of astrocytoma cells by a mechanism involving a reduction in Ca^2+^ signaling, as found for neuronal progenitors during embryogenesis [3]. Taken together, these data suggest that glutamate antagonists possess anti-cancer potential because they may promote both anti-proliferative and anti-motility effects.

How a decrease in glutamate-mediated Ca^2+^ signaling is able to reduce cell motility is an interesting question. Calcium oscillations are associated with different processes crucial for cell invasion like cell polarization [31], focal adhesion turnover [8] or regulation of metalloproteinases [32]. Many reports have shown that Ca^2+^ can alter the affinity between adhesion receptors and their specific extracellular ligands on the extracellular matrix [33-35] thereby providing a means to regulate migration. Indeed, in the presence of an intracellular Ca^2+^ chelator such as BAPTA, both human smooth muscle cells [28] and astrocytoma [8] have reduced migration. The underlying mechanisms may involve altered recycling of adhesion proteins [36] or altered disassembly of focal adhesion sites [25]. This may be due to reduced activities of Ca^2+^-dependent proteases implicated in focal adhesion protein degradation of for example, calpain [37-39] or calcineurin [40]. One of the major proteins involved in focal adhesion recycling during migration is FAK. Reduced cell motility and enhanced focal adhesion contact formation has been shown in cells from FAK-deficient mice [41]. It is now well accepted that activation of FAK promotes migration whereas inhibition of FAK or altered FAK phosphorylation decrease migration [42-46]. Several reports point out the role of glutamate receptors in the activation of FAK in a Ca^2+^ dependent manner. For example, glutamate and specific agonists of ionotropic and metabotropic glutamate receptors stimulate phosphorylation of FAK in hippocampal slices or cortical synaptosomes [47]. In high-grade glioma, AMPA receptors promotes perivascular invasion via integrins and FAK activation [48]. Moreover, glutamate stimulates phospholipase C and phosphorylation of FAK in CHO cells expressing mGluR1 receptors. Phosphorylation of FAK was reduced by PLC inhibitors or by depletion of intracellular Ca^2+^, consistent with a link between mGluR1 receptors, Ca^2+^ and FAK activation [49]. In our study, the respective order of potency of glutamate antagonists suggests that metabotropic glutamate receptors are the main receptor implicated in the Ca^2+^-dependent migration process observed in astrocytoma cells. This is not surprising in view of the role of mGluR1 in FAK activation, the major role of metabotropic glutamate receptors in astrocytes and the pattern of Ca^2+^ oscillations observed in U87MG cells which is consistent with activation of mGluR1 receptors.

Next, the question arises as to know which pool of glutamate is responsible for the enhanced migration observed in the presence of glutamate. Because migration and Ca^2+^ oscillatory behavior of these cells were dependent upon serum, it is possible that glutamate present in the serum is sufficient to account for these effects. Indeed, addition of 10% FCS in culture medium or in PBS produced a large increase in NADPH fluorescence due to formation of α-ketoglutarate, consistent with the presence of glutamate in FCS (not shown). In the presence of 10% FCS, addition of glutamate did not further enhance migration (Figure 2). Since the Ca^2+^ oscillation pattern observed during migration was quite diverse, this suggests that glutamate concentration in the cellular environment is closely regulated, probably involving controlled release and/or reuptake of glutamate. Indeed, in the presence of a glutamate reuptake inhibitor, the Ca^2+^ oscillation frequency of our cells was increased 2-fold.

The mechanism responsible for glutamate release from astrocytes has been the subject of controversy for some time. Evidence for both Ca^2+^-dependent and -independent mechanisms has been reported. The Ca^2+^-dependent mechanism is an exocytotic process similar to that observed in neurons [50,51], whereas the Ca^2+^-independant mechanism may involve swelling-dependent mechanisms [52], alteration or reversion of glutamate transporters [15,16,53] and up-regulation of the cystine-glutamate exchange system Xc^-^[15,17]. Ca^2+^-dependent release of glutamate in astrocytes represents a major pathway for intercellular communication. For example, elevation of intracellular Ca^2+^ in astrocytes was both necessary and sufficient to induce an increase in miniature postsynaptic currents in cultured hippocampal neurons, an effect prevented by the NMDA receptor antagonist AP5, consistent with release of glutamate from astrocytes [51]. Extracellular waves of glutamate were imaged during Ca^2+^ signaling in cultured astrocytes [54]. Finally, glutamate mediates calcium oscillations in astrocytes leading to the release of other transmitters like prostaglandin [55]. In our study, compounds that mobilize intracellular calcium store, like thapsigargin or t-ACPD, an agonist of the metabotropic glutamate receptors, stimulate glutamate release (Figure 3). This agrees with previous studies showing that Ca^2+^-dependent release of glutamate involves intracellular Ca^2+^ stores in astrocytes [56,57] and with the expression of metabotropic receptors in both astrocytes and astrocytomas [58,59]. Of note, in astrocytomas, glutamate release and reuptake mechanisms appear deeply altered. For example, although one of the major role of astrocytes is to protect neuron from an excess of glutamate via high capacity reuptake systems [60], astrocytomas release large amounts of glutamate which result in elevated external glutamate concetrations, up to 100 μM [14]. In our cells, the glutamate reuptake inhibitor L-THA enhanced calcium oscillations (Figure 5). As L-THA is a substrate inhibitor and therefore, being transported by the glutamate transporter in place of glutamate [61], the increase in Ca^2+^ signaling observe upon L-THA addition indicates that glutamate transporters are at least partially functional in U87MG cells. The ability of L-THA to either increase the frequency of Ca^2+^ oscillations or to induce Ca^2+^ oscillations in quiescent cells suggests that at least in part, alteration of glutamate transporters is responsible for Ca^2+^-mediated migration of astrocytoma cells.

## Conclusion

Our study uncovers an autocrine glutamate signaling loop whereby altered glutamate reuptake leads to enhanced glutamate release from astrocytoma cells and subsequent activation of glutamate receptors, particularly the metabotropic subtypes. This in turn activates calcium signaling further promoting glutamate release. Finally, Ca^2+^ oscillations induce FAK phosphorylation and focal adhesion disassembly as we already reported in this cell line [8], thus leading to enhanced migration.

## Methods

### Materials

Cell culture medium (EMEM), fetal calf serum (FCS), HEPES, L-glutamine, penicillin, streptomycin, gentamycin and trypsin-EDTA solution (0.5 g/l trypsin/0.2 g/l EDTA) were from Gibco. Glutamate, CNQX, AP3 MK801 and L-(-)-threo-3-Hydroxyaspartic acid were from Tocris. Glutamate deshydrogenase and NADP^+^ were from Sigma. Oregon Green 488 BAPTA-1 acetoxylmethylester (AM), Fura-2/AM, BAPTA/AM and Pluronic acid F-127 were from Molecular Probes.

### Cell culture

The human astrocytoma cell line U87MG was obtained from the American Type Culture Collection. Cells were maintained in 5% CO_2_ in air at 37°C in a humidified incubator on type I collagen (0.06 mg/ml) -coated plastic dishes in EMEM supplemented with 10% heat-inactivated FCS, 0.6 mg/ml glutamine, 200 IU/ml penicillin, 200 IU/ml streptomycin and 0.1 mg/ml gentamycin.

### Migration assay

U-87MG were seeded onto 35 mm diameter Petri dishes coated with Matrigel (178 μg/ml) and grown to confluence in a 37°C incubator gassed with 5% CO_2_ in air. After 24 h of serum starvation, a rectangular lesion was created using a cell scraper and cells were rinsed 3 times with culture medium containing or not 10% FCS. The cells were then incubated with the respective experimental medium supplemented or not with the compound to be tested. After 24 h of migration, 3 randomly selected fields at the lesion border were acquired using a 10x phase objective on an inverted microscope (Olympus IMT2) equipped with a CCD camera (Panasonic). In each field, the distance between the margin of the lesion and the most distant point on migrating cells was analyzed for the 10 most mobile cells. Control experiments were made in presence of vehicle, typically water or DMSO at ≤ 0.01%. Analysis was made using the *Image Tool* program (University of Texas Health Science Center at San Antonio; available by FTP from maxrad6.uthscsa.edu). For experiments with BAPTA/AM, cells were loaded for 45 min with 20 μM BAPTA/AM and 0.03% Pluronic acid F-127 in a 37°C incubator gassed with 5% CO_2_ in air prior to the creation of lesions and washing.

### Cytosolic free calcium measurements

For intracellular calcium measurements during migration, cells were cultured at subconfluence on Petri dishes in which a 2 cm diameter hole had been cut in the base and replaced by a thin (0.07 mm) glass coverslip coated with Matrigel. Experiments were performed 48 h or 72 h after plating. Cells were incubated for 45 min with the fluorescent Ca^2+^ indicator Oregon Green 488 BAPTA-1 acetoxylmethylester (5 μM) in culture medium containing 0.03% Pluronic acid F-127 in a 37°C incubator gassed with 5% CO_2_ in air. Cells were then washed twice with an external solution (in mM: 140 NaCl, 5 KCl, 2 CaCl_2_, 2 MgCl_2_, 10 HEPES and 11 glucose, pH 7.4) before Ca^2+^ measurements. Imaging was done at 30°C in external solution, with or without the compounds to be tested, using a Bio-Rad MRC-1024 laser-scanning confocal system and an inverted microscope (Nikon Eclipse) using a 40 × oil-immersion epifluorescence objective (n.a. 1.4, Nikon). Emitted fluorescence was measured at 535 ± 10 nm in response to 488 nm excitation from a krypton/argon laser, with images being usually acquired at 1 s intervals during a 15 min period. In experiments measuring intracellular calcium concentrations, cells were incubated for 30 min at 37°C in a Ringer containing 5 μM Fura-2/acetoxylmethylester (Fura-2/AM). Cells were then washed for 15 min at 37°C with Ringer solution. Digital imaging was performed at room temperature using an IMSTAR (Paris, France) imaging system. Small groups of dispersed cells were viewed using an inverted microscope (Nikon Diaphot, Tokyo, Japan) and an UV-fluor 20x objective (n.a. 0.75, Nikon). Fura-2 fluorescence was excited alternately at 340 and 380 nm, using bandpass filters (±10 nm, Nikon) and a 100 W mercury lamp (HBO, Osram). Emitted fluorescence was bandpass filtered at 510 ± 20 nm (Nikon) and measured using a Darkstar-800 CCD Camera (Photonics Sciences, Milham, UK). Acquired images were analyzed with the *fluo 210* IMSTAR software. Ratiometric Ca^2+^ images were generated at 5 s intervals, using 4 averaged images at each wavelength. After background compensation, [Ca^2+^]_i_ was averaged from pixels within manually outlined regions of interest corresponding to each cell. [Ca^2+^]_i_ values were calculated as described elsewhere (Grynkiewicz et al., 1985). Control experiments were made in presence of vehicle, typically water or DMSO at ≤ 0.01%.

### Immunocytochemistry

Cells treated or not with 20 μM BAPTA-AM for 30 min, were allowed to migrate for 24 h before immunostaining. After 15 min fixation in 4% paraformaldehyde in PBS, cells were incubated 1 h with the anti-β_1_ integrin antibody P4C10 (1/400, V/V) in PBS, and then with a FITC-conjugated goat anti-mouse secondary antibody (Zymed) for 1 h. Confocal images of migrating cells were obtained as described above, with Z-series being collected in 1 μm steps. Analysis was done after stacking the first 6 images corresponding to the basal, matrix-associated sections of the cell. The number of patches of β_1_ integrin-containing structures found at the rear of the cell was quantified in control and BAPTA-loaded cells.

### Enzymatic assay of endogenous glutamate release

Confluent U-87MG cells plated on glass cover slips were lodged in a 1 x 1 cm cuvette containing Ringer’s solution supplemented with glutamate deshydrogenase (40 U/ml) and 1 mM NADP^+^ inside a Hitachi 2000 computerized spectrofluorimeter at 37°C under stirring. Glutamate released from the preparation was immediately oxidized by GDH to α-ketoglutarate with formation of NADPH and fluorescence emission at 430 nm (delay <1 s; excitation at 335 nm). Release was quantified using standard curves constructed with exogenous addition of glutamate from 0.1 μM to 1 mM. Agents were added directly in the cuvette using a microsyringe. In experiments using BAPTA/AM, cells were first incubated for 45 min with 20 μM BAPTA/AM and 0.03% Pluronic acid F-127 in a 37°C incubator gassed with 5% CO_2_ in air, then washed twice with Ringer’s solution and placed into the cuvette. Control experiments were made in presence of DMSO at ≤ 0.01%.

### Statistical analyses

All data represent at least 3 independent experiments and results are shown as mean ± SEM. Statistical differences between two groups were determined by Student’s t-test. Analysis of variance (ANOVA) analysis was applied for multiple group comparison. Differences were considered to be significant at p ≤ 0.05.

## Abbreviations

AP3: 2-Amino-3-phosphonopropionic acid; BAPTA/AM: 1,2-*Bis* (2-aminophenoxy) ethane-*N*,*N*,*N*′,*N*′-tetraacetic acid tetrakis (acetoxymethyl ester); CNQX: 6-Cyano-7-nitroquinoxaline-2,3-dione; FAK: Focal Adhesion Kinase; L-THA: L (-)-*threo*-3-Hydroxyaspartic Acid; MAP: kinase, mitogen-activated protein kinase; MK801: (5*R*,10*S*)-(-)-5-Methyl-10,11-dihydro-5*H*-dibenzo [*a*,*d*] cylcohepten-5,10-imine maleate; NMDA: *N*-Methyl-D-aspartic acid; t-ACPD: trans (±)-1-Aminocyclopentane-*trans*-1,3-dicarboxylic acid.

## Competing interests

The authors declare no competing interests.

## Authors’ contributions

AH performed experiments. GG analyzed data and performed initial experiments. PR performed experiments, analyzed data, designed the study and wrote the paper. KT coordinated the study and participated in its design. All authors read and approved the final manuscript.
